# Buffalo Pathologic Laboratory

**Published:** 1898-06

**Authors:** 


### BUFFALO PATHOLOGIC LABORATORY.

IN SIGNING the supply bill containing an appropriation of $10,000 for the establishment at Buffalo of a State Pathologic Laboratory for the investigation into the causes and cure of cancer, Governor Black performed a service to science and the state which cannot be too highly commended. By this broadminded act the Governor has placed the state of New York on a higher scientific plane and opened way to a possible solution of one of the most vexing problems before the medical profession today.

We know there is such a thing as cancer, we know those attacked die ; but no one positively knows how, why or whence it comes, creeping along day by day, eating into the life of its victims night and day, never ending, ceaselessly progressing until finally it kills in a most horrible manner, a burdensome cross during life, a stain and a curse on the family name after death. What is more horrible to the lay mind than the sentence, "There is cancer in the family."

Theories almost without number have been advanced as to the causation of malignant tumors, but the real secret of cancerous diseases is still a mystery. At the present time the most important work doing in the direction of cancer investigation is being carried on by Prof. Sanfelice in Sardinia, and Prof. Roncali in Rome. Both are working under state aid. New York state now comes forward and extends the necessary means for American investigators to begin and carry on the work we hope to a successful issue. The laboratory will be under the directorship of Dr. Roswell Park and the preparatory work is well under way.

The fact that Buffalo, to quote from a paper on tumors recently read by Dr. Park before the American surgical association at New Orleans, "is near the center of an area some 200 miles in radius, where the death-rate from cancer is larger than any other part of the United States," makes it peculiarly fitting that the state laboratory for the study of this particular affliction should be established here.

And to Governor Black is due the thanks of the medical profession for opening the way to a thoroughly equipped investigation.

---

BARON LARREY, says the *Medical Age*, whom Napoleon I. called the "most virtuous man I have ever known," at the age of twenty-six years joined the army of the Rhine and was the physician of the so-called "flying ambulance" for twenty-two years. He was present in sixty great battles—Waterloo was one—and 400 engagements. He performed numberless operations and it is said that he did 200 amputations in one day. He operated often on the field—once took off the leg of a high officer in a driving snow storm while two others held a cloak over his patient. His name stands only below that of Ambroise Paré, his great prototype.

---

BUFFALO is the only city of any consequence in the United States which does not maintain a proper and efficient medical supervision over its public schools. It is not very long ago that the Buffalo school association made an exhaustive report on the condition of the public schools and recommended the appointment of a medical officer whose duty it should be to watch the schools and protect the health of the children.

The report was timely and to the point; and yet, in spite of the urgency of the matter, nothing has been done. Dr. Wende has made the effort, but the board of aldermen sat down on him in that extremely indifferent manner which illy became them as public officers, but which "crowns them gloriously" as politicians.

The cost of this supervision would not have appreciably affected the tax rate, yet the aldermen preferred to take chance with poorly cared for schools rather than spend $1,000 or $1,200 a year to maintain the health of many thousands of school children. But just so long as there are aldermen there will be pot-house politicians, whose ideas of public service are bounded by the line of the wards from which they are elected.

### MY SPECIALTY.

I love her; yes, I do,
Nor blush I to proclaim
That she's my specialty,
My love for her is gain.

I love her; yes, I do,
Nor make apology.
Her name, you say? ah, yes;
'Tis Jennie Cology.

---

THE medical society of the county of Erie held a special meeting Wednesday, May 11, 1898, at which its members offered their professional services to the families of the soldiers while serving their country in the field.

---

WITH the yellow flag of Spain trailing in the trough of the sea, with the scares of the yellow newspapers, it is apropos that Prof. Klebs should spring a new yellow fever bacillus on us.

---

THE common council has recommended the letting of the contract for the disposal of Buffalo's garbage to a new firm, with a new system at a ridiculously low figure. All of which is strange to an outsider, when it is considered that this new system is wholly unknown to anyone except the projectors themselves, and has been shrouded in mystery "deep as the sounding sea" and "dark as the hour 'fore dawn" even since it was first exploited.

---

AS THE May edition of the JOURNAL was entering the mails Commodore Dewey was engaging the Spanish fleet in Manila bay. In an engagement lasting but a few hours he destroyed eleven war ships and rendered *hors de combat* 618 Spaniards, with a loss on his part of only eight men slightly wounded. This is the first time in the history of the world that a fleet has been destroyed without the loss of a single man killed in the victorious squadron, and places the American commodore, who was promptly created an admiral, among the greatest naval commanders the world has produced.

